# Functional and systemic effects of whole body electrical stimulation post bariatric surgery: study protocol for a randomized controlled trial

**DOI:** 10.1186/s13063-018-2844-8

**Published:** 2018-10-31

**Authors:** Larissa Delgado André, Renata P. Basso-Vanelli, Luciana Di Thommazo-Luporini, Paula Angélica Ricci, Ramona Cabiddu, Soraia Pilon Jürgensen, Claudio Ricardo de Oliveira, Ross Arena, Audrey Borghi-Silva

**Affiliations:** 1 Department of Physiotherapy, Federal University of São Carlos, Rod. Washington Luís, km 235, São Carlos, São Paulo 13565-905 Brazil; 2 Department of Medicine, Federal University of São Carlos, Rod. Washington Luís, km 235, São Carlos, São Paulo 13565-905 Brazil; 3 Department of Physical Therapy, College of Applied Health Sciences, University of Illinois at Chicago, Chicago, IL USA; 4 Cardiopulmonary Physiotherapy Laboratory, Department of Physiotherapy, Federal University of São Carlos, Rod. Washington Luís, km 235, São Carlos, São Paulo 13565-905 Brazil

**Keywords:** Electrical stimulation, Obesity, Functional capacity, Systemic markers, Surgery

## Abstract

**Background:**

Obesity represents a major public health problem and is the fifth leading risk factor for mortality. Morbid obesity is associated with chronic systemic inflammation which increases the risk of comorbidities. Bariatric surgery (BS) is considered an effective intervention for obese patients. However, BS is associated with dietary restriction, potentially limiting physical activity. Whole-body neuromuscular electrical stimulation (WBS) could represent an innovative option for the rehabilitation of BS patients, especially during the early postoperative phase when other conventional techniques are contraindicated. WBS is a safe and effective tool to combat sarcopenia and metabolic risk as well as increasing muscle mass, producing greater glucose uptake, and reducing the proinflammatory state. Therefore, the objective of this study is to evaluate the effects of WBS on body composition, functional capacity, muscle strength and endurance, insulin resistance, and pro- and anti-inflammatory circulating markers in obese patients undergoing BS.

**Methods/design:**

The present study is a randomized, double-blind, placebo-controlled, parallel groups clinical trial approved by the Ethics Committee of our Institution. Thirty-six volunteers (body mass index (BMI) > 35 kg/m^2^) between 18 and 45 years of age will be randomized to the WBS group (WBSG) or control (Sham) group (ShamG) after being submitted to BS. Preoperative assessments will include maximal and submaximal exercise testing, body composition, blood inflammatory markers, and quadriceps strength and endurance. The second day after discharge, body composition will be evaluated and a 6-min walk test (6MWT) will be performed. The WBS or Sham protocol will consist of 30 daily sessions for 6 consecutive weeks. Afterwards, the same assessments that were performed in the preoperative period will be repeated.

**Discussion:**

Considering the important role of WBS in skeletal muscle conditioning and its value as an aid in exercise performance, the proposed study will investigate this technique as a tool to promote early rehabilitation in these patients, and as a strategy to enhance exercise capacity, weight loss, and peripheral muscle strength with positive systemic effects. The present study is still ongoing, and data will be published after its conclusion.

**Trial registration:**

REBEC, RBR-99qw5h. Registered on 20 February 2015.

**Electronic supplementary material:**

The online version of this article (10.1186/s13063-018-2844-8) contains supplementary material, which is available to authorized users.

## Background

Obesity is a chronic disease that has reached epidemic proportions in children and adults; it is currently the fifth leading risk factor for mortality [1]. According to the World Health Organization (WHO), obesity has been growing at an alarming rate in the last decade and is becoming a significant public health problem [2, 3]. Obesity is a complex multifactorial disorder associated with derangements in immune function, low-grade inflammation, and changes in the amount of pro- and anti-inflammatory cytokines [4]. Adipose tissue is a metabolically dynamic, endocrine organ that participates in the regulation of physiological processes, including immunity-related processes, through the release of pro- and anti-inflammatory cytokines [4]. These include leptin, myostatin, adiponectin, and tumor necrosis factor (TNF)-α, all of which are involved in the development of cardiometabolic diseases, such as type II diabetes, and cardiovascular disease (CVD) [4].

Adipose tissue is recognized as an active tissue able to influence food intake control, energy balance, insulin action, lipids, glucose metabolism, angiogenesis, vascular remodeling, immunity, and inflammation [3, 4]. Obesity also leads to musculoskeletal dysfunction and reduces cardiorespiratory fitness as well as muscle strength, endurance, and flexibility. These effects often significantly contribute to chronic pain and functional limitations [5].

Many patients do not respond effectively to conservative therapeutic interventions for weight reduction and require a surgical approach, which is considered safe with a low mortality rate [6–8].

Conventional gastric bypass surgery, associated with dietary restriction in the postoperative (PO) period, limits physical activity performance and can lead to reductions in skeletal muscle mass and performance [9]. As such, strategies are needed to preserve and ideally enhance functional performance. In this context, whole-body neuromuscular electrical stimulation (WBS), which differs from conventional electrical stimulation, has been recently introduced as a strategy to simultaneously stimulate several large muscle groups. It represents a promising and innovative option to enhance weight loss, improve exercise capacity and peripheral muscle strength, and induce positive systemic effects [10–12]. Several studies have demonstrated that WBS reduces sarcopenia and abdominal fat mass in both young and older sedentary individuals [13]. These studies emphasize the potential effects of WBS—being able to act beneficially when other conventional exercises are contraindicated [13].

Another study showed that WBS could, through an increase in muscle mass, potentiate the action of insulin and thus produce greater uptake of glucose in the muscle as well as reduce the proinflammatory state. These findings may be particularly relevant in the obese population which is predisposed to greater insulin resistance [14].

While WBS may hold promise as a rehabilitation strategy following bariatric surgery (BS), especially in the early stages after surgery, there appears to be no evidence about its applicability in the bariatric PO period.

Therefore, the objective of this study is to evaluate the effects of WBS on body composition, functional capacity, muscle strength and endurance, insulin resistance, and pro- and anti-inflammatory circulating markers in obese patients undergoing BS in the early PO period.

We hypothesize that WBS would enhance functional capacity, muscle strength, and endurance, and reduce inflammation in BS patients when compared with controls (Sham). Moreover, we hypothesized that WBS would reduce fat mass, improve muscle mass, and positively affect insulin resistance and exercise capacity in these patients. We also expect patients to present good acceptance and adherence to the intervention protocol.

## Methods/design

### Study design

**Fig. 1:**
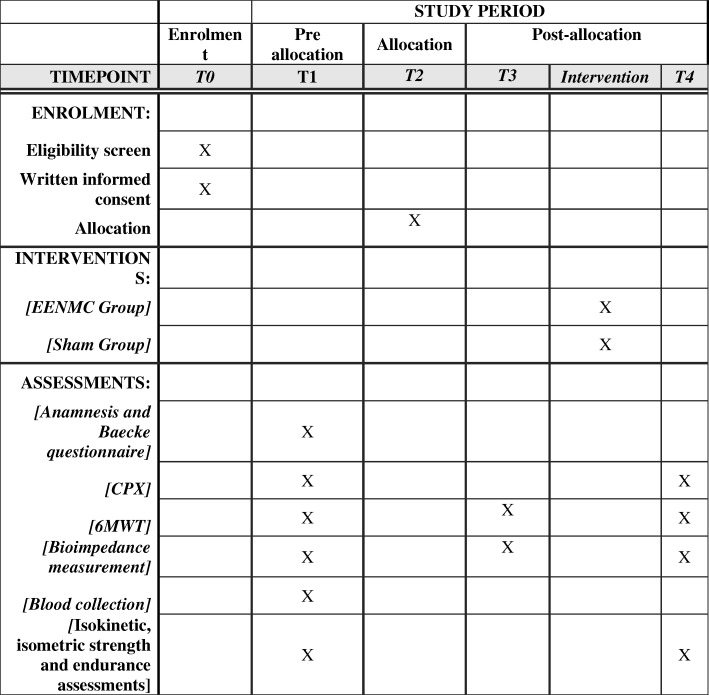
Template of content for the schedule of enrolment, interventions, and assessments. T0: baseline variables; T1: preoperative evaluations; T2: allocation; T3: postoperative evaluation; T4: postintervention evaluation. 6MWT 6-min walking test, CPX symptom-limited or maximum cardiopulmonary exercise testing, WBS (Whole-body neuromuscular electrical stimulation)

The present study is a randomized, double-blind, placebo-controlled, parallel groups clinical trial conducted at the Cardiopulmonary Physical Therapy Laboratory (LACAP) of the Federal University of São Carlos (UFSCar), São Carlos, SP, Brazil. This study was approved by the UFSCar Ethics Committee (number 966.613) and registered at the Brazilian Registry of Clinical Trials (ReBEC, RBR-99qw5h) on 20 February 2015. Recruited volunteers will all provide written informed consent prior to participation. The study will respect the Consolidated Standards of Reporting Trials (CONSORT) as show in the Fig. 1 with the template of content for the schedule of enrolment, interventions, and assessments. The study will also respect the Standard Protocol Items: Recommendations for Interventional Trials (SPIRIT) as shown in Additional file 1, respectively.

### Volunteers

#### Recruitment, randomization, and blinding

Volunteers will be recruited from among patients included in a waiting list for BS and followed by a multidisciplinary team (composed of a physician, surgeon, anesthesiologist, cardiologist, physical therapist, nutritionist, and psychologist) for a minimum period of 1 year. During this period, volunteers will be counseled on smoking and alcohol cessation and informed about the surgical procedure and PO care.

Eligible volunteers will be evaluated within 1 month before BS, 2 days after discharge and, afterwards, will be randomized to the experimental or control group with a 1:1 allocation ratio by a computer-based permuted block randomization (www.randomization.com). To achieve a gender balance for both protocols, the randomization will be stratified by gender and two randomization plans for each gender will be proposed with blocks of 4. The randomization sequence will be generated by an independent research assistant and the intervention protocol will be applied by a physical therapist. Since the present study is a double-blind clinical trial, the volunteers, the physical therapist who will perform the assessments, and the researcher who will perform the statistical analysis will be blinded to the allocation of the volunteers.

#### Inclusion and exclusion criteria

Inclusion criteria will be as follows: 1) body mass index (BMI) in class I (30–34.9 kg/m^2^), class II (BMI 35–39.9 kg/m^2^), or class III (BMI ≥ 40 kg/m^2^) obesity range for at least 3 years or more [15]. Patients with a BMI in the 30–34.9 kg/m^2^ range and presenting with associated comorbidities are candidates for BS at the discretion of medical staff and the endocrinologist [15]. Therefore, these patients will also be included in the present study); 2) no response to conventional and conservative treatment (pharmacological or not); 3) aged between 18 and 45 years; and 4) candidates for gastric bypass surgery.

Exclusion criteria will be as follows: 1) orthopedic conditions (arthrosis or pain) or neurological impairments that would preclude physical activity; 2) myocardial infarction within the last 6 months before study initiation; 3) implanted pacemaker or any metallic device; 4) unstable angina; 5) chronic heart rhythm disorders; 6) moderate or severe heart valve disease; 7) consistent history of heart disease; 8) uncontrolled hypertension; 9) uncontrolled and/or insulin-dependent diabetes mellitus; 10) β-blocker use; 11) chronic obstructive pulmonary disease (COPD) or other lung disorders; 12) any contraindication to the cardiopulmonary exercise test (CPX); 13) any health issues that compromise functional capacity testing; 14) distal arteriopathies; 15) any inflammatory, kidney, or liver conditions with documented diabetic neuropathy; 16) cognitive impairments or difficulty in comprehending or adhering to the procedures; 17) smoking; 18) use of drugs; 19) gestation; 20) use of psychotropic medications, anxiolytics, antidepressants, or appetite control medications; 21) postmenopausal women; and 22) concurrent participation in a regular exercise program. Patients with PO complications will also be excluded from this study.

#### Sample size

The sample size calculation was performed using GPower, Version 3.1.3 (Franz Faul Universität Kiel, Germany), based on a previous study by our group performed on BS patients [16] which showed a difference between the distance walked during a 6-min walking test (6MWT) before and after aerobic training of 49.7 ± 15 m. We hypothesize that we will see an improvement in 6MWT distance following WBS to a similar level as our previous study. Therefore, we projected a sample size that would provide 80% power at a significance level of α = 0.05, detecting a variation of 30% as a favorable outcome. These parameters indicate a sample of five volunteers would be required to investigate functional gains after the intervention.

The primary outcome measure for the study will be the 6MWT distance in meters since the distance walked following interventions in patients with chronic conditions is an important tool to assess the efficacy of interventions that theoretically improve functional capacity [16]. Secondary outcomes of this study are to investigate whether the intervention prevents muscle mass loss and whether there is an association between 6MWT distance with lean mass in kilograms [17]. Therefore, since the performance improvement, the prevention of lean mass loss, and the association between these measures represent important outcomes in the present study, the sample size was recalculated. Based on the bivariate normal correlation model, considering a significance level of 5%, a sample power of 95%, and a strong correlation (> 0.70) between lean mass percentage and 6MWT performance as the main outcome, the calculated sample size was 16 volunteers per group. Anticipating a dropout rate of approximately 15%, a total of 36 patients will be included in the present study.

### Procedures

Before applying the intervention protocol, volunteers will be familiarized with assessment procedures and the WBS technique. Volunteers will be evaluated in the afternoon to avoid different physiological responses due to circadian changes; they will be instructed not to drink caffeine, alcohol, or any other stimulant the night before as well as on the day of data collection, and not to perform strenuous exercises the day before data collection. All experiments will be carried out in an acclimatized room, with a controlled temperature between 22 °C and 24 °C (71.6 °F to 75.2 °F) and relative air humidity between 40% and 60%.

### Evaluations (testing procedures or measurements)

All enrolled volunteers will be submitted to anamnesis and the Baecke questionnaire will be administered to collect information about habitual physical activity related to occupation, sports, and leisure activities [18].

**Fig. 2:**
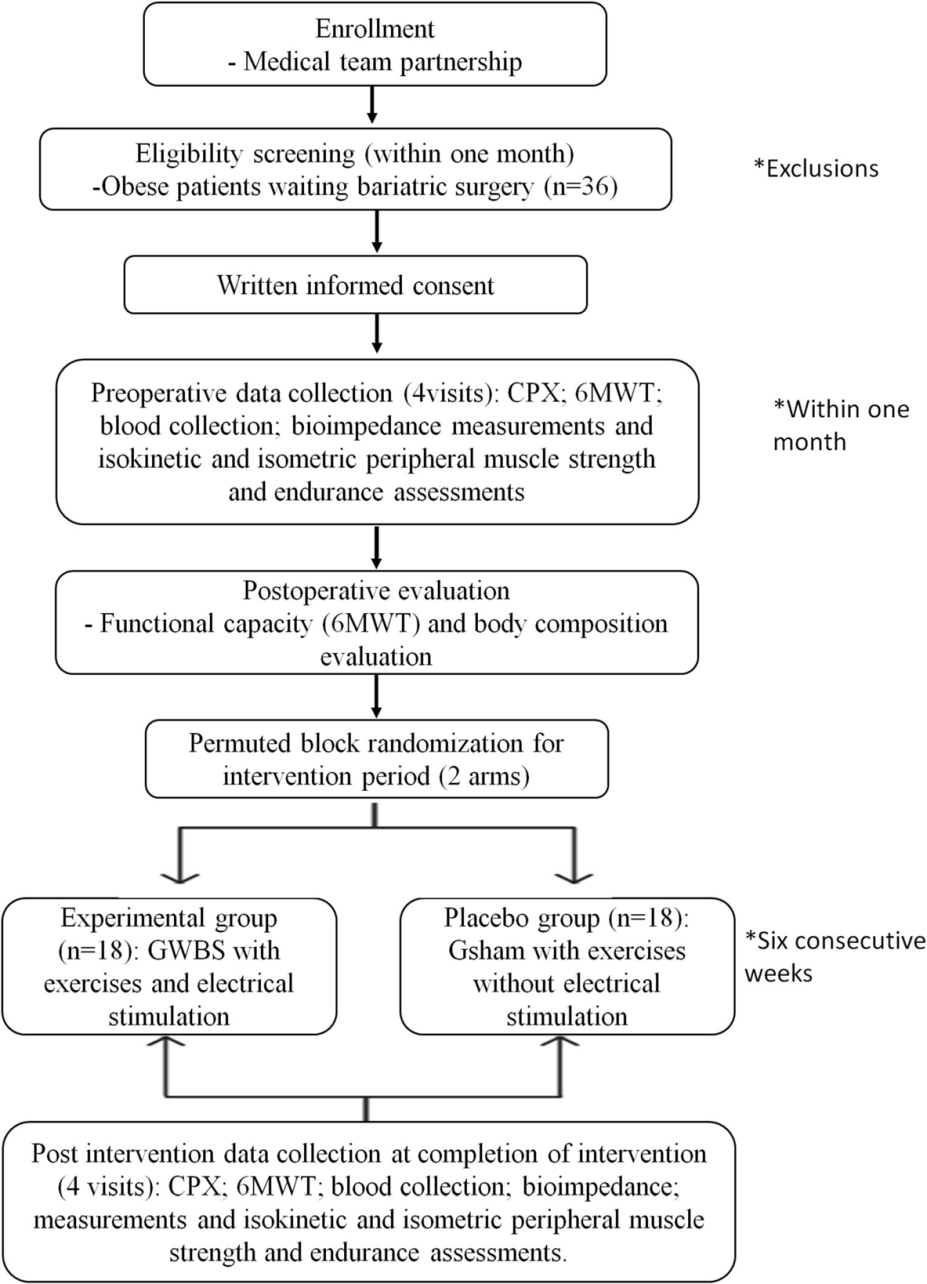
Consolidated Standards of Reporting Trials (CONSORT) flow diagram of the study. 6MWT 6-min walking test, CPX symptom-limited or maximum cardiopulmonary exercise testing

After the screening period and signing the informed consent (Additional file 2), the volunteers will return to the laboratory on 4 different days to perform the preoperative evaluations (Fig. 2), which are: 1) maximal CPX; 2) the 6MWT; 3) blood collection; 4) bioimpedance measurements; and 5) isokinetic and isometric peripheral muscle strength and endurance assessments. All evaluations will be performed again following the intervention.

The CPX will be performed on a treadmill (Super ATL, Porto Alegre, Rio Grande do Sul, Brazil) using the incremental Bruce protocol. Volunteers will be monitored by a 12-lead electrocardiogram (WinCardio System, Microme, Brasilia, Brazil) supervised by a physician, and encouraged to achieve maximal effort. Heart rate, blood pressure, and subjective responses of dyspnea and fatigue by the CR10 scale [19] will also be measured.

The 6MWT will be performed according to guidelines established by the ATS/American College of Chest Physicians [20]. The object of this test is to walk as far as possible for 6 min over a 30-m corridor. Cardiorespiratory and metabolic variables will be collected breath-by-breath through a portable Oxycon Mobile® ergospirometer (Mijnhardt/Jäger, Würzburg, Germany) throughout the 6MWT.

Blood samples will be collected the morning after a 12-h fast from the upper limb antecubital vein by a qualified professional to quantify fasting glucose levels, insulin, the insulin resistance index by the Homeostasis Model Assessment method (HOMA-IR), and the lipid profile (total cholesterol, high-density lipoprotein cholesterol (HDL-C), low-density lipoprotein cholesterol (LDL-C), very low-density lipoprotein cholesterol (VLDL-C), and triglycerides) [2, 21]. It will also be used to measure the serums concentrations of TNF-α, adiponectin, leptin, and myostatin. These samples will be analyzed by enzyme-linked immunosorbent assay (ELISA) in duplicates using high-sensitivity kits (Quantikine® HS, R&D Systems, Minneapolis, USA) according to the manufacturer’s recommendations [22]. The volunteers will be instructed not to exercise for 48 h preceding the examination and to maintain their usual diet.

Body composition will be obtained by bioelectrical impedance analysis, performed by an InBody 720 device and dual energy x-ray technique using digital absorptiometry (DXA) [23–26]. The use of both equipment for body composition will ensure all data is collected since the DXA scanning area can be limited and compromise some measures due to the larger abdominal circumference of recruited obese patients. Volunteers will be instructed to undergo a 4-h fast, to wear light clothes, not to have any metal objects in contact with their body, to urinate prior to the examination, not to drink alcohol, and not to perform strenuous exercises the day before the examination [23].

Evaluations of concentric and isometric extension of the dominant knee will be carried out using an isokinetic dynamometer (Biodex Multi-Joint System 3; Biodex Medical System, Inc., Shirley, NY) at least 48 h after the last functional test.

Isometric and isokinetic assessments will be performed using a previously described protocol [27]. The highest absolute concentric extensor and flexor torque as well as a BMI-normalized value, the flexor/extensor ratio, total work, power, and quadriceps femoris muscle fatigue at an angular speed of 60°/s will be measured [27, 28]. Volunteer positioning and the alignment of the mechanical axis, as well as the gravitational correction, will be performed as previously described [27]. Standardized encouragement will be provided by a single evaluator to stimulate volunteers to produce their maximal effort during all tests.

### Bariatric surgery

Many patients do not respond effectively to conservative therapeutic interventions and require a surgical approach. In recent years, the Roux-en-Y gastric bypass technique (RYGB) has been the most predominant modality of surgery, and is proven to be safe, leading to low mortality [6, 8]. It combines gastric restriction and reconstruction of intestinal transit through a jejunal loop that looks like the letter Y [8]. RYGB can provide greater weight loss in the long term with a reduction in several risk factors for comorbidities [8, 9].

### Postoperative data collection

In the PO period, the 6MWT and body composition measurements will be performed. These evaluations will occur within 1 week after discharge. Afterwards, volunteers will be randomized to the experimental group or the control group. At the end of the interventional trial, all preoperative evaluations will be performed a second time.

### Intervention protocol (WBS or sham protocol)

Following the PO assessments and subsequent randomization, the volunteers will return to the laboratory to perform the intervention protocol with WBS equipment (Miha Bodytec, Augsburg, Germany) [29] as described in other studies [30–32]. The experimental group (WBSG; *n* = 16) will perform the WBS protocol while the control group (ShamG; *n* = 16) will perform the same protocol without WBS stimulus.

The WBS vests and cuff electrodes allow for simultaneous control and innervation of 14–18 muscle groups or 10 regions (upper legs, upper arms, bottom, abdomen, chest, lower back, upper back, including the latissimus dorsi) with a selectable intensity for each one allowing a total electrode area of up to 2800 cm^2^ [32, 33]. The strain and current intensity can be individually selected and modified during the protocol and be progressively increased during each training session. The parameters will be saved on smart cards to ensure reliable and valid application during the WBS protocol [31, 32].

**Fig. 3:**
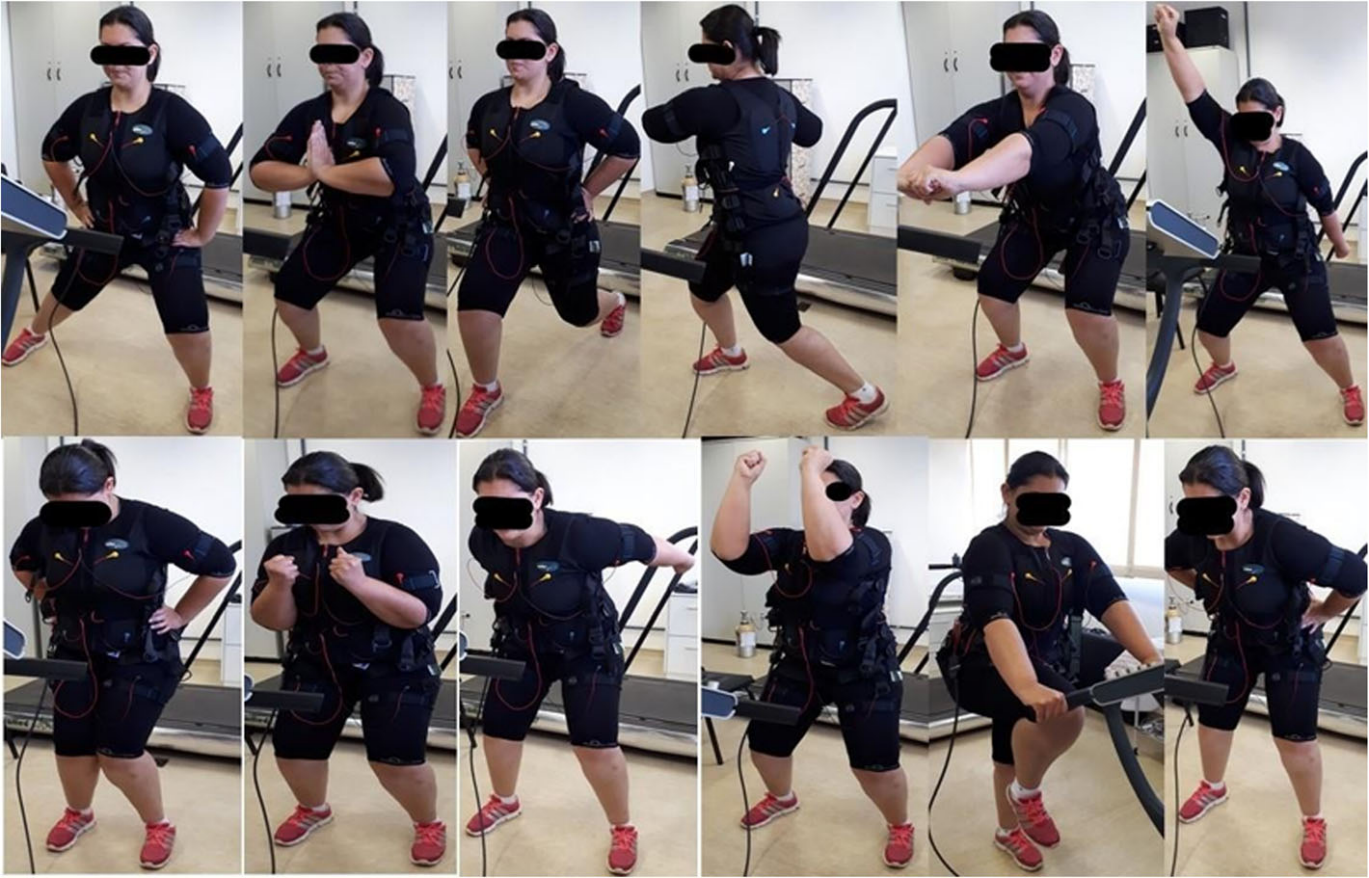
Whole-body electrical stimulation exercises performed by a recruited volunteer

WBS stimulation will be accompanied by low-intensity movements to allow an effective contraction (maximum voluntary contraction requested by the supervisor) during electrical stimulation. The exercises will be carried out without any additional weights with volunteers in the standing position. Specifically, WBS will be performed in conjunction with 10–14 dynamics: squat, trunk flexion, exercises of upper limbs, and isometric abdomen contraction. In the experimental group, these muscle contractions will be performed simultaneously with an electrical stimulation current. Figure 3 illustrates a volunteer performing the protocol with a WBS vest.

The sessions will last approximately 20–30 min using a previously described protocol, involving a rectangular wave by a symmetrical bipolar electric pulse [13]. Considering a total of 6 weeks of training for both groups, the WBS protocol will be performed in three nonconsecutive days of endurance alternating with 2 days of training per week. All sessions will occur with the supervision of a trained physical therapist.

The endurance training session will apply a bipolar electrical current with a frequency of 85 Hz and an impulse breadth of 350 μs intermittently with 6 s of electrical stimulation using a direct impulse boost to perform the exercise and 4 s off for rest, with ramp of 0.4 s. The total time under current per session will be approximately 12 min, with approximately 5 min of rest between sets. The strength sessions will consist of the same bipolar electrical current, with a frequency of 30 Hz and impulse breadth of 350 μs intermittently, 4 s of electrical stimulation and 10 s of rest, with ramp of 0.4 s. These sessions will last approximately 8 min, with approximately 5 min of resting between exercises.

The intensity of both sessions is prescribed to reach submaximal levels, which will be adjusted to each region according to disparities of current sensitivity, respecting visual and effective contraction, without volunteer discomfort [30, 31]. To maintain an adequate and tolerable exercise intensity, the BORG scale was used. Thus, volunteers should score between 2 and 3 for the perception of effort in this scale in the endurance protocol and above 5 in the protocol of force [31, 32].

The difference between the WBS and control intervention is that the intensity of electrical stimulation will be switched off in the latter group to avoid any influence of WBS equipment during the exercises. Volunteers will be instructed not to perform any regular exercise activities, being questioned weekly with respect to compliance with these recommendations. After the postintervention assessment, the WBS protocol will be offered to the control group.

### Postintervention data collection

All preoperative assessments will be performed at the end of the intervention period. At the end of the protocol and assessments, all volunteers will receive a report with all data evaluations.

### Statistical analysis

A blinded researcher will perform all statistical analyses for this study. The volunteers will be codified by the researcher responsible for randomization to ensure blinding of control and intervention groups. Descriptive statistics including the mean, standard deviation (SD), and quartiles will be applied to summarize the data. The Shapiro Wilk test will be used to investigate data distribution. Data will be expressed as mean, SD, and 95% confidence interval. The primary analysis is to assess the effect of the intervention on 6MWT distance and the secondary analysis is the relationship between 6MWT distance and the lean mass loss.

In this sense, the effect of WBS will be assessed in the context of the following hypotheses:

- H0: WBS does not improve 6MWT distance and/or does not prevent the loss of muscle mass.
- H1: WBS increases 6MWT distance and/or prevents the loss of muscle mass.

If the data present a normal distribution, analysis of variance (ANOVA) will be tested for comparisons between the body composition and 6MWT distance change considering three periods of evaluation (preoperative, postoperative, and postintervention). In instances where data are not normally distributed, nonparametric tests will be applied.

The benefits obtained by both groups will be demonstrated through the difference between postintervention results minus PO data with all variables that will be collected. Comparisons between groups will be assessed using the unpaired Student *t* test or Mann-Whitney test, as appropriate.

Effect size will be analyzed using Cohen’s *d*, and the results will be interpreted based on the Cohen classification [34] as follows: small (0.21–0.49), medium (0.50–0.79), or large (> 0.80).

The statistical analysis using correlation will assess the degree of association (or dependence) between improvements in functional tests, body composition data (InBody 720 and DXA), muscular assessment (dynamometry), and concentration of cytokines.

Appropriate correlation tests will be used if normality of distribution, linearity, variability, and homoscedasticity is satisfied from the scatter diagram performed a priori. The correlations will be defined as weak (0.20–0.50), strong (0.50–0.70), and very strong (> 0.70) [35]. The statistical analysis will be performed using SPSS Statistics for Windows, Version 17.0. (SPSS Inc., Chicago, IL, USA) and MedCalc, version 11.4.4.0 (MedCalc Software, Mariakerke, Belgium), adopting a significance level of 5%. Per-protocol and intention-to-treat analyses will be conducted.

## Discussion

Among bariatric surgical techniques, the RYGB has brought satisfactory long-term results such as increased weight loss and subsequent maintenance, with a concomitant reduction in several risk factors in bariatric patients. In this sense, previous investigations have demonstrated it is a more effective procedure compared with other surgical approaches [36, 37]. Some longitudinal studies observed that different isolated bariatric surgical techniques have a positive influence on reducing weight at 6 and 12 months, improving functional capacity, and even reducing comorbidities associated with obesity [38–40]. However, it has recently been demonstrated that the inclusion of structured physical activity in the short term (within 4 months of the surgical procedure) can also result in functional capacity improvement [41, 42].

On the other hand, most studies in which physical exercise have been proposed after BS results in considerable attrition with low adherence to exercise rehabilitation and weight-loss programs [15, 43–46]. Another important feature is the severe dietary restriction especially in the first 3 months after surgery that is recommended during the early PO period [9]. This stage involves a reduction in protein intake which affects fat but also lean mass leading to accelerated weight loss [47]. In the early PO period, patients submitted to gastric bypass surgery should start a liquid diet on the first day and maintain this diet for the first week following surgery [48]. Associated with this, patients have a surgical rest period with a restriction on moderate to high-intensity exercise [49]; pain is also a frequent issue following surgery. Thus, promoting physical activity in this patient population following surgery is a challenge [9].

In this context, intervention strategies achievable as soon as possible after the surgical procedure could have a positive impact in this population. Current evidence has shown that WBS may be an alternative and potentially beneficial approach associated with conventional physical exercise programs in different populations [12].

A contemporary study demonstrated that WBS positively effects sarcopenia and abdominal body fat parameters across the lifespan and in sedentary volunteers [13]. This research highlights the potential effects of this electromyostimulation technique and its beneficial impact on body composition and functional capacity which may overcome some limitations of conventional physical exercise programs, especially when it is contraindicated [13]. Another recent study indicated that a WBS program was feasible and effective for elderly volunteers, being a favorable alternative for improving body composition and physical strength in postmenopausal and overweight women [11, 33].

Furthermore, positive effects of WBS on muscle parameters have been previously observed in athletes [10], healthy young individuals [49], the elderly, and patients with chronic disease [50, 14]. Additionally, other studies have shown that WBS could increase muscle mass, enhance insulin action and, thus, induce a greater glucose uptake into skeletal muscle [51]. These findings may be of particular relevance, especially in obese individuals who are predisposed to higher levels of insulin resistance [14]. A recent study found that WBS application to the abdomen for 12 weeks was effective in reducing a proinflammatory state (as observed by TNF-α, adiponectin, and C-reactive protein levels) [51], as well as fasting glycemia, insulin resistance, and visceral adiposity in obese diabetics [5]. However, the potential effects of this technology on inflammatory markers after BS are unknown and warrant further study.

Additionally, it is important to emphasize that WBS equipment can penetrate subcutaneous tissue and stimulate deeper muscles due to the ability to select high current intensities. Thus, WBS can be considered an innovative and promising technique in obese individuals who have difficulty performing exercise, emphasizing the importance of this study.

Data will be published after completion of the study.

### Trial status

Patient recruitment is currently underway.

## Additional files

Additional file 1: SPIRIT 2013 checklist: recommended items to address in a clinical trial protocol and related documents. (DOC 122 kb)

Additional file 2: Model consent form. (DOCX 16 kb)

## Abbreviations

6MWT: Six-minute walking test
BMI: Body mass index
BS: Bariatric surgery
COPD: Chronic obstructive pulmonary disease
CPX: Cardiopulmonary exercise test
CVD: Cardiovascular disease
DXA: Dual energy x-ray technique using digital absorptiometry
ELISA: Enzyme-linked immunosorbent assay
HDL-C: High-density lipoprotein cholesterol
LDL-C: Low-density lipoprotein cholesterol
PO: Postoperative
RYGB: Roux-en-Y gastric bypass technique
ShamG: Control group
TNF: Tumor necrosis factor
VLDL-C: Very low-density lipoprotein cholesterol
WBS: Whole-body neuromuscular electrical stimulation
WBSG: Whole-body neuromuscular electrical stimulation group
WHO: World Health Organization
