# Supplementary material for: Functional and systemic effects of whole body electrical stimulation post bariatric surgery: study protocol for a randomized controlled trial
Source: Trials. 2018 Oct 31;19:597. doi: 10.1186/s13063-018-2844-8 (PMC6211515; doi:10.1186/s13063-018-2844-8)
Supplement: Supplementary file 2 — Model consent form. (DOCX 16 kb) [file 13063_2018_2844_MOESM2_ESM.docx]

**TERMO DE CONSENTIMENTO LIVRE E ESCLARECIDO**

Consentimento de participação no trabalho: Efeitos funcionais, clínicos e sistêmicos da estimulação elétrica corpórea no pós-operatório de cirurgia bariátrica.

Responsáveis:

## Profa. Dra. Audrey Borghi e Silva – Departamento de Fisioterapia - UFSCar

Pos-doutoranda: Luciana Di Thommazo Luporini– Programa de Pós-graduação em Fisioterapia – UFSCar

Eu, _______________________________________, RG N.º ________________ e CPF _________________, declaro estar ciente das condições sob as quais me submeterei ao trabalho acima citado, proposto pela Prof^a^. Dra. Audrey Borghi e Silva. O trabalho tem como objetivo principal verificar a existência de associação entre variáveis obtidas por testes de avaliação funcional e muscular de forma não invasiva e coleta sanguínea venosa de forma invasiva por profissional qualificado e delegado para tal função.

Inicialmente serei submetido a uma avaliação física, um teste cardiopulmonar máximo em esteira ergométrica, sob supervisão médica e fisioterapêutica e entrevista e, caso selecionado, realizarei uma avaliação de constituição física por bioimpedância elétrica, uma avaliação de força muscular em equipamento apropriado para este fim e testes funcionais em campo (testes de caminhada e subida e descida de degraus), sob supervisão dos profissionais responsáveis pelo estudo. Após a cirurgia, serei reavaliado e participarei de um programa de exercícios leves junto com a eletroestimulação, que é um tratamento para auxiliar no fortalecimento dos músculos e poderá auxiliar na perda de peso. O tratamento poderá causar desconforto, e este será reduzido com a dosagem corrigida, de acordo com a minha tolerância.

Estou ciente de que minha participação no presente estudo envolve mínimo risco, uma vez que os testes são de fácil execução e possuem alta aplicabilidade clínica, sendo amplamente utilizados, além de serem acompanhados pelos profissionais responsáveis integralmente. Adicionalmente, as devidas medidas de segurança serão tomadas. Os testes propostos consistem em modalidades de caminhada em terreno plano, subida de degrau único de 20 cm de altura e caminhada em esteira ergométrica com todos os aparatos de segurança necessários para a execução dos mesmos e avaliação de força muscular em equipamento bastante seguro (dinamômetro isocinético), sendo que o mesmo também possui dispositivo de segurança para interrupção do teste quando necessário e solicitado a qualquer instante. No início, durante e após os testes serão monitorizadas variáveis fisiológicas, de modo que será medida minha pressão arterial pelo método auscultatório indireto, frequência cardíaca e questionada minha percepção subjetiva ao esforço e qualquer alteração serei avisado pelo fisioterapeuta e o teste será interrompido imediatamente. Além disso, durante o teste será analisado o comportamento do meu coração, pelo uso de um equipamento semelhante a um relógio, sem a utilização de drogas medicamentosas ou de procedimentos invasivos. Serei orientado(a) a comunicar os avaliadores sobre eventuais sintomas, tais como tontura, turvação visual, náuseas, dor, cansaço e fadiga, que eu possa vir a apresentar para que providências adequadas sejam tomadas.

No teste cardiopulmonar máximo, realizado previamente aos demais testes e acompanhado pelo profissional médico, serei monitorado por meio da derivação MC5, no qual será avaliado o comportamento eletrocardiográfico com relação a possíveis alterações isquêmicas e à existência bem como quantidade, se houver, dos seguintes tipos de arritmias: extra-sístole isolada, ventricular unifocal ou multifocal, bloqueio divisional, fibrilação atrial, bloqueio completo de ramo direito, pausa sinusal e taquicardia ventricular não sustentada antes, durante e após o teste.

Na ocasião de ocorrerem riscos de qualquer natureza, quais sejam, quedas e/ou demais acidentes, lesões osteomioarticulares, mal-estar e/ou instabilidade hemodinâmica, os próprios pesquisadores se responsabilizam pelas condutas de primeiros socorros, bem como encaminhamento médico quando necessário e/ou qualquer tipo de avaliação fisioterapêutica como resultado de dano físico.

Ressalta-se que somente farei parte do trabalho se estiver de acordo com critérios estabelecidos para um grupo de indivíduos cujas características serão determinadas por meio de uma avaliação detalhada, sendo selecionado(a) apenas se estiver em condição clinicamente estável.

Além disso, sei que minha participação nesse estudo é estritamente voluntária e, portanto, não receberei qualquer forma de remuneração pela minha participação no experimento, podendo desistir de participar da pesquisa a qualquer momento sem dano ou prejuízo. Por fim, sei que os dados obtidos desse trabalho serão mantidos em sigilo e não poderão ser consultados por outras pessoas sem minha autorização por escrito, ao menos para fins científicos, resguardando, portanto, minha privacidade.

Eu li e entendi todas as informações contidas neste documento, assim como as da Resolução 196/96 do Conselho Nacional de Saúde.

São Carlos, _______ de _____________________ de 2017.

______________________________

Assinatura do Voluntário

Responsáveis

Profa. Dra. Audrey Borghi e Silva Pós-doutoranda: Luciana Di Thommazo Luporini
